# Interaction of Type 1 Porcine Reproductive and Respiratory Syndrome Virus With *In Vitro* Derived Conventional Dendritic Cells

**DOI:** 10.3389/fimmu.2021.674185

**Published:** 2021-06-02

**Authors:** Yanli Li, Enric Mateu

**Affiliations:** ^1^ Departament de Sanitat i Anatomia Animals, Facultat de Veterinària, Universitat Autònoma de Barcelona, Cerdanyola del Vallès, Spain; ^2^ IRTA, Centre de Recerca en Sanitat Animal (CReSA, IRTA-UAB), Bellaterra, Spain

**Keywords:** PRRSV1, conventional dendritic cells, TLR3, TLR7, innate immune response

## Abstract

The present study delineates the interaction of a typical PRRSV1.1 isolate 3267 (moderate virulence) with *in vitro* derived pig conventional dendritic cells, cDC1, cDC2, and a CD14^+^ population (designated as CD14^+^ DCs). cDC1 and cDC2 were not susceptible to 3267 infection, but a fraction of CD14^+^ DCs were infected. After exposure to the virus, all three DC types remained immature as determined by no increase of maturation molecules (MHC-I, MHC-II, CD80/86, CCR7), no release of cytokines, no modification of antigen presentation abilities, and no alteration of endocytic/phagocytic capabilities. However, when infected MARC-145 cells were used as a source of viral antigens, cDC2 and CD14^+^ DCs showed a significant increase in the expression of maturation molecules and substantial release of cytokines, notably IL-12/IL-23p40 (by both DC types) and IL-10 (by CD14^+^ DCs). To address the impact of PRRSV1 3267 on TLR3- and TLR7-mediated activation, cDC1, cDC2, and CD14^+^ DCs were inoculated by the virus (live or UV-inactivated) for 6 h prior to or simultaneously with the addition of poly I:C (TLR3 ligand) or gardiquimod (TLR7 ligand; not used for cDC1). Compared with using TLR ligand alone, combination with the virus did not result in any alteration to the maturation markers on all DC types but changed the cytokine response to either TLR3 or TLR7 ligand. Pre-exposure of cDC2 or CD14^+^ DCs to the live virus resulted in an increased production of IFN-α upon poly I:C stimulation, while pre-exposure to UV-inactivated virus tended to enhance the release of IL-10 upon gardiquimod stimulation. Simultaneous addition of the live virus and the TLR ligand either had no effect (mainly in cDC2) or impaired most of the cytokine release after gardiquimod stimulation (in CD14^+^ DCs). When used as antigen presenting cells, cDC2 pre-inoculated by the live virus before addition of gardiquimod impaired the proliferation of CD4^–^CD8^–^ T cells. In the case of CD14^+^ DCs, pre-exposure to the live virus or simultaneously added with TLR3 or TLR7 ligand largely decreased the proliferation of CD4^–^CD8^+^ and CD4^–^CD8^+^ T-cell subsets. For cDC1, no significant changes were observed in cytokine responses or T-cell proliferation after poly I:C stimulation. Of note, cDC1 had a short life during *in vitro* culturing, for which the results obtained might be biased. Overall, exposure to PRRSV1 did not induce maturation of cDC1, cDC2, or CD14^+^ DCs, but modified TLR3 and TLR7-associated responses (except for cDC1), which may affect the development of adaptive immunity during PRRSV1 infection. Moreover, the sensing of infected cells was different from that of the free virus.

## Introduction

*Porcine reproductive and respiratory syndrome viruses* (PRRSV1 and PRRSV2) are positive-stranded RNA viruses, classified in the Family *Arteriviridae*, Order *Nidovirales*. Since the emergence in 1980s, they spread rapidly to most pig-producing countries, causing significant economic losses to the swine industry. The infection produces respiratory disease in piglets and reproductive failure in pregnant sows; it could also produce asymptomatic infections in animals with some level of previous immunity or when the infecting strain is of low virulence (1–4). For both PRRSV1 and 2, infected animals are able to clear the virus and develop a solid homologous immunity, although this happens after a long viremic period (weeks to months) and several weeks more with the virus detected in lymphoid tissues (5).

The immune response of pigs to PRRSV is unusual with a delayed development of neutralizing antibodies (NAb) and an irregular cell-mediated response during the first weeks of infection (6–8). For more complexities, PRRSV viremia was found in the presence of neutralizing antibodies (9); moreover, it could be resolved before the development of neutralizing antibodies (10). Such features suggest that PRRSV may deceive the immune system. Interaction with dendritic cells (DCs) could be one of the mechanisms of deception.

DCs are specialized in sampling antigens and launching immune response by conveying differentiation signals to naive CD4^+^ T cells (11–13); they thus are presumed to be the central players in orchestrating adaptive immune responses during viral infection. Before identification and characterization of *bona fide* DCs in pigs, bone marrow- (BMDCs) and monocyte-derived dendritic cells (moDCs) were widely employed to study the interaction of PRRSV with DCs. Although with controversies, most of the studies showed that the virus may dysregulate DCs by preventing DC maturation and the production of cytokines (14–20). Also, some reports indicated PRRSV-exposed DCs may induce regulatory T cells (Tregs) (21). But it must be noted that BMDCs and moDCs represent models of inflammatory DCs (22); thereby, the results obtained would be biased from what may happen in the *in vivo* environment.

With the access to conventional (c) cDC1, cDC2, and plasmacytoid DCs (pDCs), the interaction of *bona fide* DCs with PRRSV can be better studied. pDCs isolated from blood were used to determine type I IFN responses to PRRSV. Little or no inhibition of type I IFN responses were detected except for highly virulent PRRSV2 strains (23). Recently, the study of Nazki et al. (24) indicated that respiratory cDCs were recruited at the peak of PRRSV viremia. Bordet et al. (25) using *ex vivo* lung cDCs demonstrated cDC1, cDC2 were not susceptible to PRRSV1, and that the highly virulent PRRSV1.3 Lena induced a higher Th1 polarization compared to PRRSV1.1 strains.

Although the *ex vivo* approach is highly representative of what happens in the infected animals, the scare number of DCs obtained from lymphoid tissues, blood, or lungs makes it difficult to proceed with in-depth functional studies. For that, a reliable alternative model for *in vitro* studies is demanded. The Flt3 ligand (Flt3L)-stimulated *in vitro* derivation model was recently validated for generating *in vivo* equivalent cDC1 and cDC2 as well as an unclassified CD14^+^ population from bone marrow hematopoietic cells (26). In the present study, we used this model to explore the impact of a typical PRRSV1.1 strain on maturation, cytokine production, and antigen presentation abilities of the three generated DC populations, called cDC1, cDC2, and CD14^+^ DCs for the present study. The use of infected cells as a source of antigens and interference of PRRSV1 with TLR3- and TLR7-mediated cDC activation were also studied.

## Materials and Methods

### Obtention of Bone Marrow Hematopoietic Cells and Alveolar Macrophages, and Isolation of Peripheral Blood Mononuclear Cells

Bone marrow hematopoietic cells (BMHCs) were aseptically obtained from the femora and humeri of 4-week-old pigs. Briefly, bones were cut into approximately 1 cm^3^ pieces and agitated in PBS at room temperature (RT) for at least 1 h. The resulting cell suspension was filtered through a 70 μm strainer before being depleted of erythrocytes by lysis with 0.15 M NH_4_Cl solution. Finally, cells were filtered through a 40 μm strainer, washed, and resuspended in a freezing medium (90% FBS, 10% dimethyl sulfoxide [DMSO]) (Sigma-Aldrich, Spain). Cells were frozen in liquid nitrogen until used.

Alveolar macrophages (AMs) were collected by broncho-alveolar lavage. In brief, lungs were removed from euthanized pigs and then were filled by pouring phosphate-buffered saline (PBS) into the trachea. After a gentle massage, the bronchoalveolar lavage was collected and centrifuged. AM pellets were washed with PBS and finally frozen as described for BMHCs.

PBMCs were isolated from the blood of 12-week-old pigs by density gradient centrifugation using Histopaque 1.077. Cells were frozen using CryoStor cell cryopreservation media (Sigma-Aldrich, Spain) in liquid nitrogen.

Donor animals were euthanized using approved methods according to Spanish (Royal Decree 53/2013) and European Union regulations (Directive 2010/63). Animals were sedated before euthanasia with a pentobarbital overdose. All donor pigs were negative of PRRSV, *porcine circovirus type* 2 (PCV2), *Mycoplasma hyopneumoniae*, *torque teno sus virus* (TTSuV) 1 and 2, and *influenza A virus* as determined by Real-Time quantitative PCR (RT-qPCR). Batches of BMHCs, AMs, and PBMCs used had viabilities > 90% after thawing as assessed by trypan blue (Sigma-Aldrich, Spain) staining. For PBMCs, the capability of T-cell proliferation was also verified. Cells from four animals were used.

### Virus Production and Titration

PRRSV1.1 isolate 3267 (Genbank accession n° JF276435) was used in the present study. This strain has been extensively used previously (23, 27–30). Strain 3267 was propagated in AMs and used as a sixth passage (used in all experiments except otherwise indicated). There were not cytokines (IFN-a, IFN-g, IL-4, IL-6, IL-12/IL-23p40, IL-10, IL-1b, TNF-a) detected in 3267 produced in AMs. The titre reached 7.2 log10 TCID50/ml, whereby only a low volume was needed for a specific multiplicity of infection (MOI) in the following experiments. In parallel, strain 3267 was adapted to MARC-145 cells and used as a third passage (named MARC3267). Titration of the produced viruses was performed on AMs or MARC-145 according to the system where the virus was initially propagated. Titres were calculated using the Reed-Muench method (31). The endpoint of infection in cell cultures was identified by immunofluorescent staining of PRRSV1 nucleocapsid (N) with a specific antibody (clone 1C5H; Ingenasa, Spain).

Virus inactivation was performed by exposing the viral suspensions to a UV-254 source at a dose of 200 mJ/cm^2^. Inactivation was validated by titration of the UV-treated virus on AMs or MARC-145 cells according to the system where the virus was initially propagated.

### Generation and Sorting of DCs

DCs were produced using Flt3L as described previously (26). Briefly, BMHCs were seeded in the 24-well plates (non-treated; Corning, Spain) at a density of 1 × 10^6^ cells/600 µl in RPMI 1640 medium (Sigma-Aldrich, Spain) containing 20 ng/ml recombinant human Flt3L (rhuFlt3L, Fisher Scientific, Spain), 10% of fetal calf serum (FCS), 2mM glutamine, 20mM HEPES, 100 units/ml penicillin, and 100 μg/ml streptomycin. Cells were cultured for 14 days, with half of the medium replaced every three days.

DCs were stained for CADM1/MHC-II/CD172a/CD14 as reported before (26). Fluorescence minus one (FMO) controls, were used for gating analysis. Primary antibodies (conjugated or not) and their working dilutions are listed in **Table 1**. cDC1 (CADM1^+^CD14^–^MHC-II^hi^CD172a^–/lo^), cDC2 (CADM1^+^CD14^–^MHC-II^hi^CD172a^+^), and CD14^+^ DCs (CADM1^+^CD14^+^MHC II^hi^CD172a^+^) were sorted on the BD FACSJazz sorter (BD Biosciences, Oxford, UK). The other flow cytometric analyses were acquired on the MACSQuant Analyzer 10 (Miltenyi Biotec, Bergisch Gladbach, Germany). Data were examined using the FCS Express 7 software (*de novo* Software, Glendale, CA, United States).

**Table 1:** Antibodies used for flow cytometry analysis.

Antibody	Clone	Isotype	Species produced	Working dilution	Supplier
Anti-human CADM1	3E1	IgY	Chicken	1/1000	MBL
Anti-CD14 FITC conjugated	MIL2	IgG2b	Mouse	1/100	Bio-Rad
Anti-CD172a	BL1H7	IgG1	Mouse	1/500	Bio-Rad
Anti-SLA II DR	2E9/13	IgG2b	Mouse	1/500	Bio-Rad
Anti-CD11R1	MIL4	IgG1	Mouse	1/500	Bio-Rad
Anti-CD11R3	2F4/11	IgG1	Mouse	1/500	Bio-Rad
Anti-CD1	76-7-4	IgG2a	Mouse	1/50	Bio-Rad
Anti-CD163	2A10/11	IgG1	Mouse	1/250	Bio-Rad
Anti-CD3	PPT3	IgG1	Mouse	1/200	Bio-Rad
Anti-CD25	K231.3B2	IgG1	Mouse	1/80	Bio-Rad
Anti-CD4 FITC conjugated	74-12-4	IgG2b	Mouse	1/200	BD Pharmingen
Anti-CD8a Alexa Fluor 647 conjugated	76-2-11	IgG2a	Mouse	1/100	BD Pharmingen
Anti-human CCR7 (CD197) PE-Cy7 conjugated	3D12	IgG2a	Rat	1/20	BD Pharmingen
Anti-DEC-205 (hybridoma supernatant)	9HZF7	IgG1	Mouse	1/1	Provided by Lab. de Inmunología, CIAD, A.C.
Anti-MHC-I (hybridoma supernatant)	4B7/8	IgG2a	Mouse	1/10	Gift from Dr. J. Domínguez*
human CD152 (CTLA-4)-muIg fusion protein	–	IgG2a	Mouse	1/50	Ancell
Anti-mouse/rat FoxP3	FJK-16s	IgG2a	Rat	1/40	eBioscience

Antibodies without specific indications are anti-pig antibodies.

*Department of Biotechnology, INIA, Madrid, Spain.

### PRRSV Infection

In the first experiment, unsorted DCs (500,000 cells) were inoculated by PRRSV1 3267 at MOI 0.1 for 1.5 h (5% CO2, 37°C) in the 15 ml tubes (cap loosed). Unbound virus was washed away, and a fresh RPMI 1640 medium was added (containing 10% FCS, now onwards referred to as complete RPMI). Cells were dispensed in the flat-bottomed 96-well plates with a final volume of 200 μl. At 48 h post-inoculation (hpi), cell cultures were collected and subject to a three-color staining for PRRSV1, CD14, and a third molecule (MHC-II, DEC205, CD163, CD172a, CD11R1, or CD11R3). Briefly, cells were incubated with primary antibodies of the third molecules, followed by Alexa Fluor 647-conjugated secondary antibodies (anti-mouse IgG1 or IgG2a). Then, the anti-CD14-FITC antibody was added. PRRSV1 was detected by using an RPE conjugated (Bio-Rad, Spain) PRRSV1 N specific antibody (clone 1C5H; Ingenasa, Spain) after cells were fixed (4% paraformaldehyde) and permeabilized (0.3% saponin).

In the second experiment, sorted cDC1, cDC2, or CD14^+^ DCs (50,000 cells) were infected as described above. At 48 hpi, supernatants were collected and titrated in AMs (as described above). Cells were stained for PRRSV1 N protein as described above and examined on a flow cytometer.

### Stimulation of cDC1, cDC2, and CD14^+^ DCs

Sorted cDC1, cDC2, and CD14^+^ DCs were dispensed in the round-bottomed 96-well plates at a density of 15,000 cells/well for cDC1 and 30,000 cells/well for cDC2 and CD14^+^ DCs. Then, cells were inoculated with PRRSV1 3267 at an MOI of 10 or incubated with complete RPMI for 6 h. After that, poly I:C (10 μg/ml, to all DC types) or gardiquimod (10 μg/ml, to only cDC2 and CD14^+^ DCs) (both from InvivoGen, Spain) was added to the cultures. With additional 18 h, DCs were harvested, and the viability was determined by Near-IR dead cell staining kit (Fisher Scientific, Spain). Expression of MHC-I, MHC-II, CD80/CD86, and CCR7 was examined by flow cytometry. For CD80/86, a dimeric protein with the extracellular domain of human CD152 fused to murine IgG2a Fc was used. CCR7 was labeled by an antibody anti-human CCR7 (CD197), which has been verified to cross-react with porcine lymphocytes (32). Alexa Fluor 647-conjugated anti-mouse IgG2a (Fisher Scientific, Spain) was used as the secondary antibody for MHC-I and CD80/86 staining. The referred antibodies are listed in **Table 1**. DCs were also used to perform mixed lymphocyte reaction as described below in the “*Mixed Lymphocyte Reaction Assay*” subsection.

### Co-Culture of cDC1, cDC2, and CD14^+^ DCs With Infected MARC-145 Cells

Two days prior to co-culture, MARC-145 cells were infected with MARC3267 (MOI 0.1), then detached and 10:1 mixed with cDC1, cDC2, or CD14^+^ DCs at a final concentration of 1.1 × 10^6^/ml for cDC2 and CD14^+^ DC, and 0.55 × 10^6^/ml for cDC1 (a final volume of 200 μl in the 96-well round-bottomed plates). DCs co-cultured with mock-infected MARC-145 cells or with only complete RPMI were used as negative controls; DCs stimulated with poly I:C was used as the positive control. After 24 h of incubation, DCs were labeled for MHC-I, MHC-II, CD80/86, and CCR7 as described above. Cell culture supernatants were frozen at –80°C until cytokine assessment. To establish the dose-response curve, an increasing proportion of infected cells was generated by infecting MARC-145 cells with different doses of MARC3267. Infection was quantified by flow cytometry examination of PRRSV1 N.

To examine the role of apoptotic cells in DC activation, MARC-145 cells were exposed to UV radiation (312 wavelength) and then cultured for 4 h at 37°C 5% CO_2_ to develop apoptosis. A radiation dose (dose 300 mJ/cm^2^) that generated an apoptosis profile resembling 3267-infected MARC-145 cells (MOI 0.01, 48 hpi) were used to co-culture with cDC2 or CD14^+^ DCs. The apoptosis was assessed by Annexin V and propidium iodide (PI) staining. DCs exposed to plain medium, mock-infected MARC-145 cells, or supernatants from infected or mock-infected MARC-145 cells were used as negative controls. DCs exposed to poly I:C (10 μg/ml) were used as the positive control. At 24 h, the expression of MHC-I, MHC-II, CD80/86, and CCR7, and the antigen presentation ability (as described below in “*Mixed Lymphocyte Reaction (MLR) Assay”*) were determined.

### Mixed Lymphocyte Reaction Assay

cDC1, cDC2, and CD14^+^ DCs that have been exposed to different stimuli were washed to remove the remaining virus, poly I:C, or gardiquimod. Then they were 1: 5 mixed with allogeneic PBMCs that were labeled with CellTrace Violet. After 5 days of incubation, cells were harvested and stained for CD3, CD4, and CD8a.

For determining the frequency and proliferation of Tregs, only DCs exposed to viral suspensions were examined. In this case, half of the supernatant of the original DC culture was kept with cells for mixing with PBMCs. After 5 days, cells were collected and incubated with an anti-CD25 antibody (Bio-Rad, Spain) biotinylated by EZ-Link Sulfo-NHS-SS-Biotin (Fisher Scientific, Spain); then, streptavidin PerCP-Cy5.5 was added followed by a CD4a-FITC antibody. Foxp3 intracellular staining was performed with an anti-Foxp3 PE antibody using the Foxp3/Transcription Factor Staining Buffer Set (eBioscience, United States). Proliferation was determined by examining the intensity of CellTrace Violet fluorescence.

### Endocytosis/Phagocytosis Assay

cDC2 and CD14^+^ DCs were inoculated with PRRSV1 3267 at an MOI of 10 and incubated for 24 h at 37°C, 5% CO_2_. Then, cells were centrifuged, seeded at a density of 10,000 cells/well in round-bottomed 96-well plates, and pulsed with dextran-FITC (molecular weight 40,000; Sigma-Aldrich, Spain) at 1 mg/ml or Alexa 488-labeled *S. aureus* (Fisher Scientific, Spain) at 20 μg/ml (equivalent to 60 particles/cell) for 2.5 h and 1 h, respectively. Cells were incubated either at 37°C or on ice (negative control). Free Dextran-FITC was washed away. Endocytosis of dextran was determined by comparing the median fluorescence intensity (MFI) of cells incubated at 37°C with the MFI of cells incubated on ice. Extracellular bound *S. aureus* was quenched by the addition of 1 mg/ml trypan blue. The phagocytic capability was determined by examining the proportion of AF488-*S. aureus* positive cells as published before (26).

For examining the phagocytosis of infected cells, cDC2 or CD14^+^ DCs were co-cultured (1 h, 37°C) with PRRSV1 3267-infected MARC-145 cells (ratio of 1:5) that were labeled with CellTrace Violet (Fisher Scientific, Spain). Alveolar macrophages, the natural target of PRRSV, were not used because they are also phagocytes and secret substantial amount of pro-inflammatory cytokines (TNF-α, IL-1β). Cells incubated on ice or co-cultivated with mock-infected MARC-145 cells were used as controls. DCs (MHC-II^+^) harboring Violet fluorescence were considered as positive of phagocytosis.

### Detection of Cytokines/Chemokines

The presence of cytokines/chemokines IFN-α, IFN-γ, IL-4, IL-6, IL-12/IL-23p40, IL-10, IL-1β, TNF-α, and IL-8 (CXCL8) in cell culture supernatants was determined using Porcine ProcartaPlex Multiplex Immunoassay (Invitrogen, Spain) according to the manufacturer’s instructions. Cytokines/chemokines were captured by fluorescent antibody-coated beads, thereafter identified by adding 25 μl of biotinylated detection antibodies followed by 50 μl of streptavidin-PE antibodies. TGF-β1 was measured by Simplex ProcartaPlex Immunoassay (Invitrogen, Spain). Cell culture supernatants were acid treated to activate TGF-β1 before adding to the antibody-coated beads. Results were quantified on a MAGPIX system using a four-parameter logistic regression curve. All samples were run in duplicate.

### Statistical Analysis

All data were analyzed using the GraphPad Prism 9.0 software package (GraphPad Software, La Jolla, CA, United States). Statistical tests applied to each data set are indicated in figure legends.

## Results

### cDC1 and cDC2 Were Not Susceptible to PRRSV1 Infection, a Fraction of CD14^+^ DCs Were Infected

**Figure 1:**
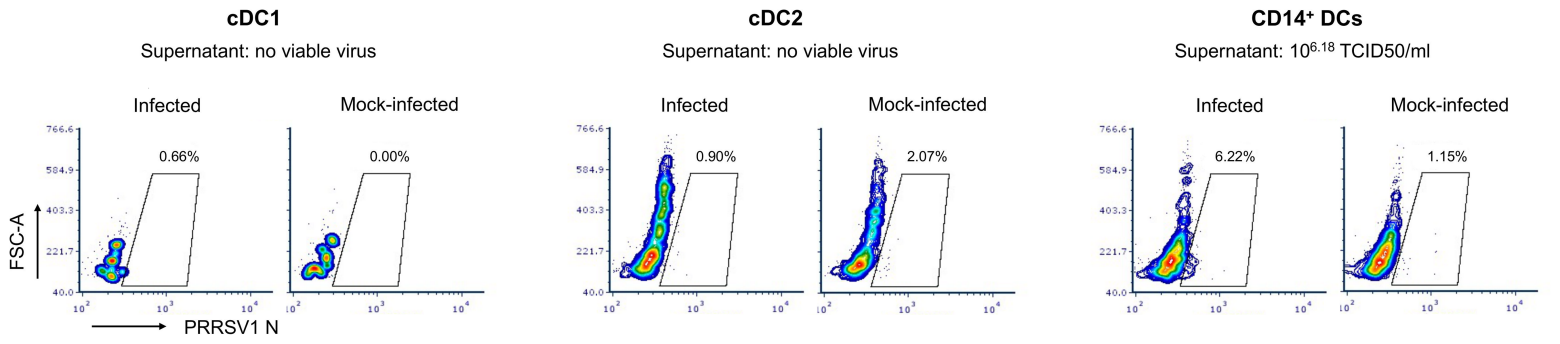
Susceptibility of cDC1, cDC2, and CD14+ DCs to PRRSV1 3267 infection. Cells were infected by PRRSV1 3267 at MOI of 0.1 for 48 h. The infection was determined by flow cytometry staining of PRRSV1 nucleocapsid (N) protein and titrating supernatants in alveolar macrophages (AMs). Mock-infected cells were used as the negative control (mock-infected). Results were obtained with cells from two pigs. The contour plots from one pig are shown as the representative.

cDC1, cDC2, and CD14^+^ DCs were sorted and infected with PRRSV1 3267 at an MOI of 0.1 for 48 h. As shown in **Figure 1**, PRRSV1 N protein labeling was only detected in a small but consistent fraction of CD14^+^ DCs (6.2 ± 1.3%). The supernatants of the infected CD14^+^ DCs contained high viral titers, on average 6.2 ± 0.8 log_10_ TCID_50_/ml. No virus was detected in cDC1 or cDC2 (**Figure 1**).

Parallel infection of the unsorted DCs showed that all N^pos^ cells were CD14^–^ (**Supplementary Figure S1A**). The proportion of CD14^+^ cells decreased significantly in the inoculated cultures compared with the mock-inoculated ones (30.7 ± 5.0% versus 18.2 ± 3.7%, respectively, *p*<0.05; 33.2 ± 2.8% before infection) (**Supplementary Figure S1B**). The proportion of cells harboring other cell surface markers were not significantly changed. Infected cells were further characterized as MHC-II^+^ CD172a^+^ CD163^+/–^ DEC205^–^ CD11R1^+/–^ CD11R3^+/–^ CD1^+^ (**Supplementary Figure S1C**).

### Exposure of CDC1, CDC2, and CD14^+^ DCs to PRRSV1 3267 Suspensions Did Not Change the Expression of MHC-I, MHC-II, CD80/86, or CCR7, Did Not Induce any Significant Cytokine Release, Did Not Modify T-Cell Proliferation in the MLR, and Did Not Affect the Endocytic/Phagocytic Capabilities

**Figure 2:**
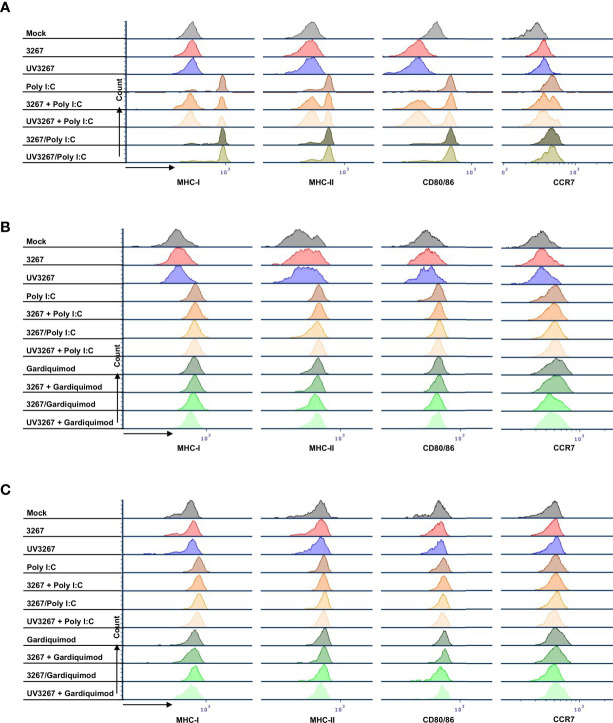
Maturation of cDC1, cDC2, and CD14^+^ DCs. DCs were inoculated with PRRSV1 3267 (MOI 10), live or UV-inactivated (UV3267), for 6 h prior to (for example, 3267+Poly I:C) or simultaneously with (for example, 3267/Poly I:C) poly I:C (10 μg/ml, to cDC1, cDC2, and CD14^+^ DCs) or gardiquimod (10 μg/ml, to only cDC2 and CD14^+^ DCs). Plain medium (complete RPMI 1640) was used as the negative control (mock) in each step. After incubation for extra 18 h, cells were harvested and stained for MHC-I, MHC-II, CD80/CD86, and CCR7 on cDC1 **(A)**, cDC2 **(B)**, and CD14^+^ DCs **(C)**. Data of cDC1 were obtained from two pigs, while cDC2 and CD14^+^ DCs were from four pigs. The histograms shown are from one pig as the representative.

**Figure 2** shows the result of the flow cytometry assessment of MHC-I, MHC-II, CD80/86, and CCR7 expression on cDC1, cDC2, and CD14^+^ DCs after incubation with PRRSV1 3267 (MOI 10, 24 h). As shown, exposure to the virus did not induce a significant increase in the expression of the mentioned molecules compared to DCs cultured with plain medium. Results were similar when using MOI 1.0 (data not shown).

**Figure 3:**
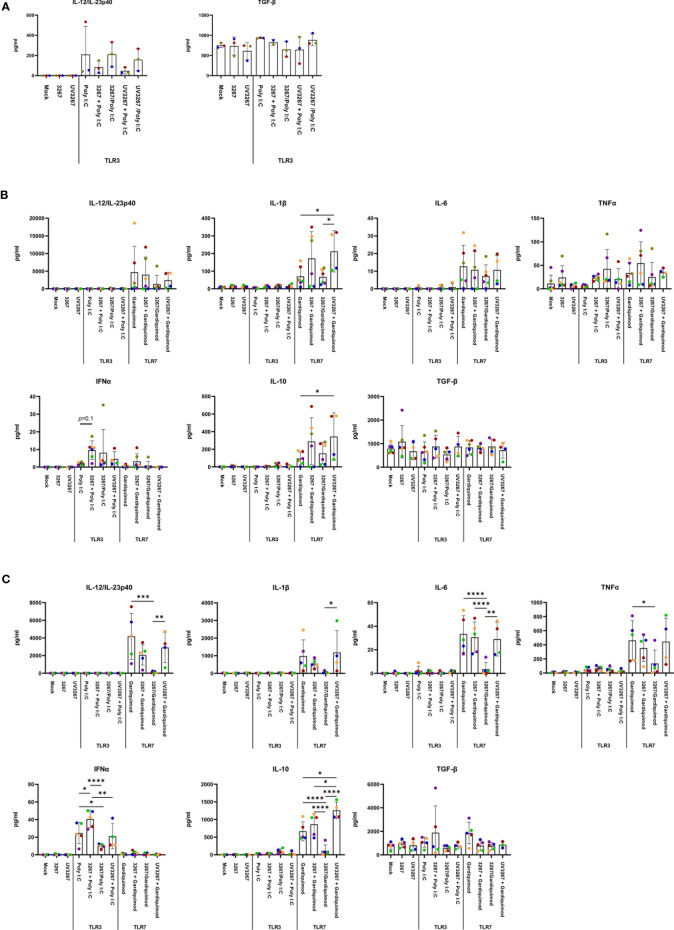
Cytokine production by cDC1, cDC2, and CD14^+^ DCs. DCs were inoculated with PRRSV1 3267 (MOI 10), live or UV-inactivated (UV3267), for 6 h prior to (for example, 3267+Poly I:C) or simultaneously with (for example, 3267/Poly I:C) poly I:C (10 μg/ml, to cDC1, cDC2, and CD14^+^ DCs) or gardiquimod (10 μg/ml, to only cDC2 and CD14^+^ DCs). In each step, plain medium (complete RPMI 1640) was used as the negative control (mock). After additional 18 h, cytokines (IFN-α, IFN-γ, IL-4, IL-6, IL-12p40, IL-10, IL-1β, TNF-α, and IL-8 (CXCL8)) in the supernatants were assessed by a multiplex immunoassay, and TGF-β was assessed by a simplex immunoassay. Production of IL-12/IL-23p40 and TGF-β (the only two cytokines detected) in cDC1 **(A)**, and IL-12/IL-23p40, IL-1β, IL-6, TNF-α, IFN- α, IL-10, and TGF-β in cDC2 **(B)** and CD14+ DCs **(C)** are shown. IFN-γ and IL-4 are not shown as they were rarely detected; IL-8 (CXCL8) is not shown as its standard was not good enough to use. The values are displayed as bars with mean and standard deviation indicated. Symbols in different colors represent different animals; symbols in red and orange are DCs from the same pig but derived in different days, the same as blue and bright green symbols. Symbols in the same color throughout **(A–C)** represent the same animal. Results of cDC1 were obtained from three animals, while cDC2 and CD14^+^ DCs were from four animals. Statistical significance was calculated by the Kruskal Wallis test with Tukey test for multiple comparisons. ****p < 0.0001, ***p < 0.001, **p < 0.01, and *p < 0.05.

Exposure to the virus did not induce any significant release of IL-12/IL-23p40, IL-10, IFN-α, IL-6, IL-1β, TNF-α, IFN-γ, or IL-4 (**Figure 3**). When UV-inactivated PRRSV1 3267 (referred to as UV3267 below) was used, the result was similar, suggesting that the viability of the virus was not relevant to these variables. As the positive controls, exposure of DCs to poly I:C or gardiquimod induced an evident increase in the expression of MHC-I, MHC-II, CD80/86, and CCR7, as well as the release of cytokines for all DC types (**Figures 2** and **3**). Of note, the viability of cDC2 and CD14^+^ DCs did not drop after exposure to the virus for 24 h compared with exposure to plain medium. However, the viability of cDC1 dropped significantly if they were not stimulated by poly I:C, which maintained the viability of cDC1 up to 70.8 ± 3.2% after 24 h of incubation. When exposed to the virus or plain medium, less than 50% of cDC1 were viable at 6 h and much less (< 10%) at 24 h.

**Figure 4:**
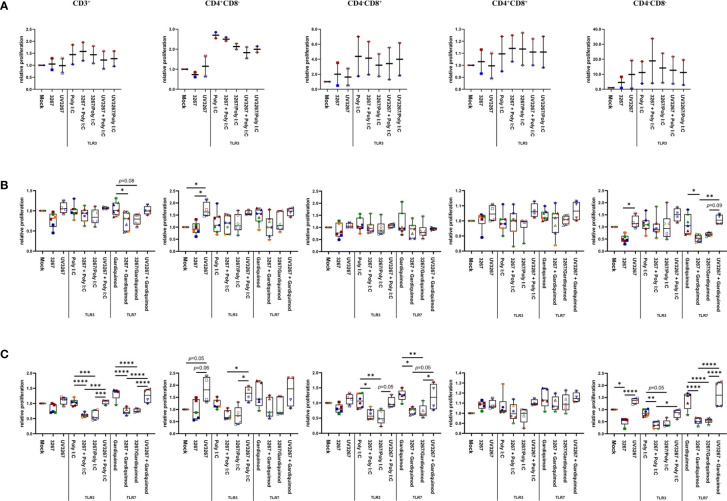
Proliferation of T cells induced by cDC1, cDC2, and CD14^+^ DCs. cDC1 **(A)**, cDC2 **(B)**, and CD14^+^ DCs **(C)** were inoculated with PRRSV1 3267 (MOI 10), live or UV-inactivated (UV3267), for 6 h prior to (for example, 3267+Poly I:C) or simultaneously with (for example, 3267/Poly I:C) poly I:C (10 μg/ml, to cDC1, cDC2, and CD14^+^ DCs) or gardiquimod (10 μg/ml, to only cDC2 and CD14^+^ DCs). The cultures were incubated for additional 18h. Plain medium (complete RPMI 1640) was used as negative control (mock) in each step. Such generated DCs were mixed with allogeneic pig PBMCs (labeled with CellTrace Violet dye) at a ratio of 1:5. After 5 days, cells were harvested and stained for CD3, CD4, and CD8α. Proliferation of CD3^+^, CD4^+^CD8α^–^, CD4^–^ CD8α^+^, CD4^+^CD8α^+^, and CD4^–^CD8α^–^ T cells was determined by CellTrace Violet dilution by flow cytometry. Gating strategy was CD3^+^ → CD4^+^CD8α^–^, CD4^–^ CD8α^+^, CD4+CD8α^+^, CD4^–^CD8α^–^ → proportion of diluted Violet (**Supplementary Figure S3**). In each group, one symbol represents DCs from one pig; symbols with the same color in the same box mean DCs were from the same pig but derived in different days; symbols with the same color throughout **(A–C)** represent the same animal. Data are shown as boxplots (25th–75th interquartile range), with median and whiskers showing minimum to maximum datapoints. For each type of DCs, proliferation induced by DCs cultured with plain medium (mock) was considered as 1.0, and the other populations were normalized to it. Data was normalization based on TLR3- or TLR7-related groups without cross-normalization. Data are from three independent experiments with two animals for cDC1 and four animals for cDC2 and CD14^+^ DCs. Statistical significance was calculated by the Kruskal Wallis test with Tukey test for multiple comparisons. ****p < 0.0001, ***p < 0.001, **p < 0.01, and *p < 0.05.

Virus-stimulated cDC1, cDC2, and CD14^+^ DCs were further used as antigen-presenting cells in an MLR. cDC1 pre-incubated with either live or inactivated virus were not efficient in sustaining T-cell proliferation as expected by the damaged cell viability. For cDC2 and CD14^+^ DCs, exposure to the live virus did not cause any significant effect on global T-cell proliferation, although some impairment was observed in the proliferation of the CD4^–^CD8^–^ subset (**Figure 4**). By contrast, the use of UV3267 resulted in a significantly higher (*p* < 0.05) proliferation rate of CD4^+^CD8^–^ cells for both cDC2 and CD14^+^ DCs, and CD4^–^CD8^–^ for CD14^+^ DCs.

The endocytic and phagocytic capabilities of cDC2 and CD14^+^ DCs was also examined. Exposure to the virus did not modify the capacity of cDC2 or CD14^+^ DCs to capture soluble dextran-FITC or particulate Alexa 488-labeled *S. aureus* (**Supplementary Figure S2**). cDC1 were not included here because of the low number of available cells.

### Pre-Exposure of cDC2 or CD14^+^ DCs, but Not cDC1 to PRRSV1 3267 Suspensions Enhanced Proliferation of CD4^+^CD25^+^Foxp3^+^ Regulatory T Cells (Tregs)

**Figure 5:**
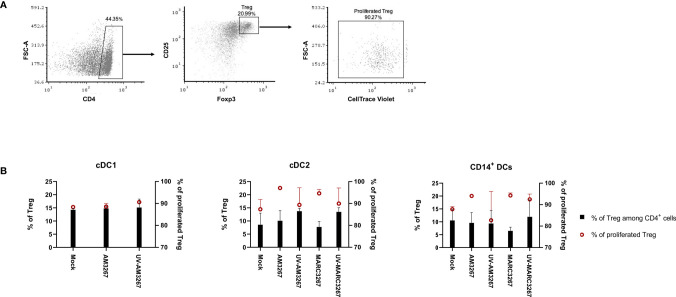
Proportion and proliferation of Tregs induced by PRRSV1-stimulated cDC1, cDC2, and CD14^+^ DCs. DCs were inoculated by live and UV-inactivated PRRSV1 3267 (MOI 10) that was propagated in either AMs or MARC-145 cells for 24 h, then mixed with allogeneic pig PBMCs (labeled with CellTrace Violet) at a ratio of 1:5. After 5 days, cells were harvested and stained for CD4, CD25, and Foxp3. DCs incubated with plain medium (mock) were used as the negative control. **(A)** Cells labeled as CD4^+^CD25^+^Foxp3^+^ were defined as Tregs, among which Violet-diluted cells were considered as proliferated Tregs. **(B)** The proportion of Tregs among CD4^+^ cells (black bars) and the proliferation of Tregs (red symbols) were shown with a dual Y-axis graph. Data are from three animals for each DC type.

Next, we examined the capacity of DCs to induce Tregs after exposure to PRRSV1. To this end, cDC1, cDC2, and CD14^+^ DCs were exposed to PRRSV1 3267, then mixed with allogeneic PBMCs in an MLR assay. The proportion of CD4^+^CD25^+^Foxp3^+^ Tregs and their proliferation rate were determined. As shown in **Figure 5**, exposure of DCs to the virus did not increase the frequency of Tregs but enhanced their proliferation. The enhancement occurred with virus propagated in both AMs and MARC-145 cells, but not with UV-treated virus. Of note, exposure of cDC2 to UV3267 resulted in a higher proportion of Tregs (**Figure 5**). Examination of IL-10 and TGF-β in the supernatants of MLR cultures did not find any difference between cDC2 or CD14^+^ DCs exposed to the virus and to the plain medium (data not shown).

### PRRSV1 Modified the Response of cDC2 and CD14^+^ DCs to Poly I:C and Gardiquimod Stimulation

To further investigate the impact of PRRSV1 3267 on cDC1, cDC2, and CD14^+^ DCs, we examined whether exposure to PRRSV1 could modify TLR3- and TLR7-mediated activation. To this end, cDC1, cDC2, and CD14^+^ DCs were exposed to viable or UV-inactivated virus for 6 h prior to or simultaneously with poly I:C (TLR3 agonist) or gardiquimod (TLR7 agonist) stimulation.

Since cDC1 are not responsive to gardiquimod stimulation (26), only TLR3-mediated activation was examined. As a result, PRRSV1 3267 did not affect the maturation of cDC1 (**Figure 2A**) or the production of IL-12/IL-23 p40 (**Figure 3A**), the only responsive cytokine of cDC1 upon poly I:C stimulation, regardless of 3267 was added before or simultaneously with poly I:C. In the following MLR assay, all combinations of virus and poly I:C resulted in similar T-cell proliferation values (**Figure 4A**).

For cDC2, pre-exposure to the virus did not modify the expression of cell surface maturation markers compared with the use of poly I:C or gardiquimod alone (**Figure 2B**). Regarding cytokine release, pre-exposure to the live virus resulted in an evident release of IFN-α upon poly I:C stimulation, although the amount was not significant compared with poly I:C was added alone (*p*=0.1) (**Figure 3B**). Gardiquimod stimulation produced a wide panel of cytokines. The production of IL-1β and IL-10 was enhanced (*p*<0.05) when cDC2 were pre-incubated with UV-treated virus. This was not observed when live virus was used. The exposed cDC2 were then used as antigen-presenting cells in an allogeneic MLR assay. As shown in **Figure 4B**, pre-exposure of cDC2 to the live virus before TLR7 activation resulted in an impaired proliferation of CD4^–^CD8^–^ T cells. This effect was not observed when UV3267 was used. No changes were observed when the virus was combined with poly I:C (TLR3-mediated activation).

For CD14^+^ DCs, pre-exposure to live 3267, but not UV3267, enhanced IFN-α release upon addition of poly I:C (*p*<0.05), while simultaneous addition of the live virus and poly I:C strongly decreased the production of IFN-α (*p*<0.05). This downregulation was also observed for IL-12, IL-6, TNF-α, and IL-10 when CD14^+^ DCs were exposed to the virus and gardiquimod at the same time (**Figure 3C**). Of note, the highest induction of IL-10 was obtained by pre-exposing CD14^+^ DCs to UV-3267 followed by TLR7 activation (*p*<0.05). In the MLR assay, significant inhibition of T-cell proliferation, particularly in CD4^–^CD8^+^ and CD4^–^CD8^–^ subsets, was induced by CD14^+^ DCs that were exposed to the virus prior to or simultaneously with TLR7 activation (**Figure 4C**). A similar inhibition effect was also found in combination with TLR3 activation. The inhibition was not observed when the UV-treated virus was used.

TGF-β was not induced by the virus or the combination with any TLR ligand above levels in the control samples (**Figure 3**).

### cDC2 and CD14^+^ DCs, but Not cDC1 Efficiently Sensed 3267-Infected Cells

**Figure 6:**
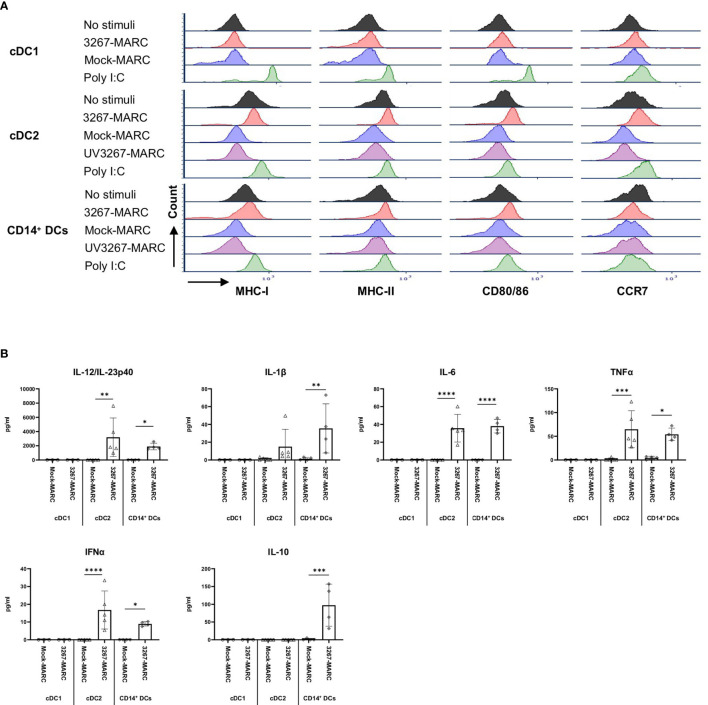
Sensing of infected MARC-145 cells by cDC1, cDC2, and CD14^+^ DCs. cDC1, cDC2, and CD14^+^ DCs were co-cultured with MARC-145 cells that were infected with live or UV-inactivated PRRSV1 3267 that was propagated in MARC-145 (3267-MARC and UV3267-MARC, respectively; UV3267-MARC were not used for cDC1). DCs stimulated by poly I:C or cultured with plain medium were used as the positive and negative controls, respectively. After 24 hours, **(A)** DC maturation was determined by flow cytometry staining of MHC-I, MHC-II, CD80/86, and CCR7. **(B)** Cytokine production was assessed by a multiplex immunoassay for IFN-α, IFN-γ, IL-4, IL-6, IL-12p40, IL-10, IL-1β, TNF-α, and IL-8 (CXCL8). Cytokines in the co-culture of DCs and UV3267-MARC were not examined and are not shown; rarely detected IFN-γ and IL-4 and unworkable IL-8 (CXCL8) are also not shown. Data are obtained from three animals for cDC1 and four animals for cDC2 and CD14^+^ DCs. Statistical significance was calculated by the Kruskal Wallis test with Tukey test for multiple comparisons. ****p < 0.0001, ***p < 0.001, **p < 0.01, and *p < 0.05.

We then investigated the response of different DC types to PRRSV1-infected cells. For that purpose, we exposed cDC1, cDC2, and CD14^+^ DCs to 3267-infected MARC-145 cells (62.6 ± 1.2% of PRRSV1 N^+^ cells). As shown in **Figure 6A**, the expression of MHC-I, MHC-II, CD80/86, and CCR7 on cDC2 was significantly increased (*p* < 0.05), in contrast to co-culture with mock-infected cells or plain medium. For CD14^+^ DCs, the expression of MHC-I, MHC-II, and CD80/86 was also increased but not that of CCR7 (**Figure 6A**). MARC-145 cells inoculated with UV-treated virus were unable to activate neither cDC2 nor CD14^+^ DCs.

Responsiveness of cDC2 and CD14^+^ DCs to infected MARC-145 cells was also reflected in cytokine/chemokine production. While co-culture of cDC2 with mock-infected MARC-145 cells did not induce any significant secretion of cytokines, the use of infected cells resulted in a substantial release of IL-12/IL-23p40 (2231.3 ± 762.0 pg/ml) along with lower quantities of IFN-α, IL-6, and TNF-α (**Figure 6B**). CD14^+^ DCs exposed to infected MARC-145 cells had a similar IL-12/IL-23p40 response but also produced IL-10 (147.0 ± 14.4 pg/ml) (**Figure 6B**). In contrast, cDC1 were not responsive to infected MARC-145 cells in any of the performed tests (data not shown).

**Figure 7:**
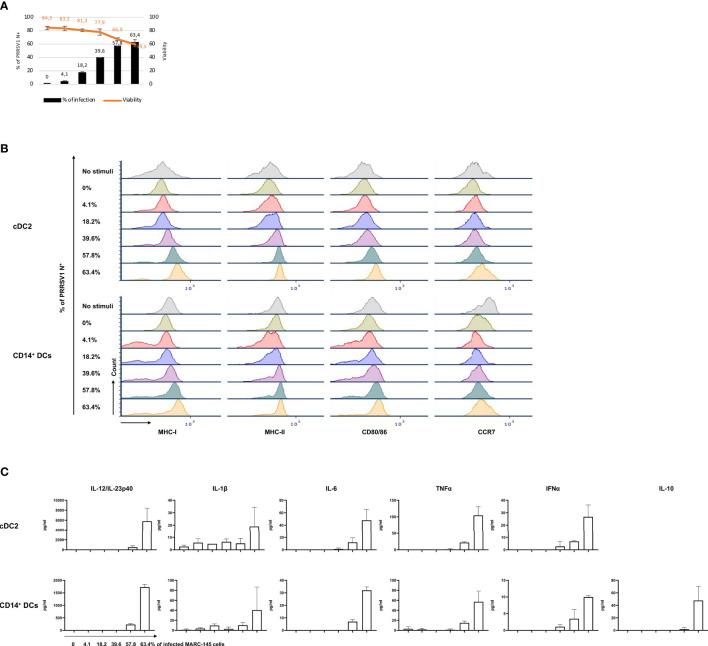
Response of cDC2 and CD14^+^ DCs to different doses of PRRSV1 3267 infected MARC-145 cells. **(A)** An increasing % of infected (PRRSV1 N^+^) MARC-145 cells (black bars) was generated by infecting MARC-145 cells with different doses of MARC3267. The viability was indicated upper (orange line); **(B)** Expression of MHC-I, MHC-II, CD80/86, and CCR7 in cultures of cDC2 (upper) and CD14^+^ DCs (lower) exposed to MARC-145 cells containing different % of infected cells (indicated at the left of Y-axis); **(C)** Cytokine production in cultures of cDC2 (upper) and CD14^+^ DCs (lower) exposed to MARC-145 cells containing an increasing % of infected cells (indicated below IL-12/IL-23p40 of CD14^+^ DCs). IL-10 was not detected in cDC2, thus not shown.

To further confirm the observed effect was attributable to the infection of MARC-145 or its consequences, a dose-response experiment was performed. cDC2 and CD14^+^ DCs were co-cultured with MARC-145 cells that displayed increasing proportion of infected cells (PRRSV1 N^+^), on average 0%, 4.1 ± 1.1%, 18.2 ± 0.7%, 39.6 ± 0.8%, 57.8 ± 0.2%, 63.4 ± 3.1%, respectively (**Figure 7A**). The results showed that phenotypical maturation (**Figure 7B**) and cytokine production (**Figure 7C**) of cDC2 or CD14^+^ DCs were evident only when the infection reached at a higher level, > 39.6 ± 0.8%, where the cell viability dropped significantly (**Figure 7A**).

**Figure 8:**
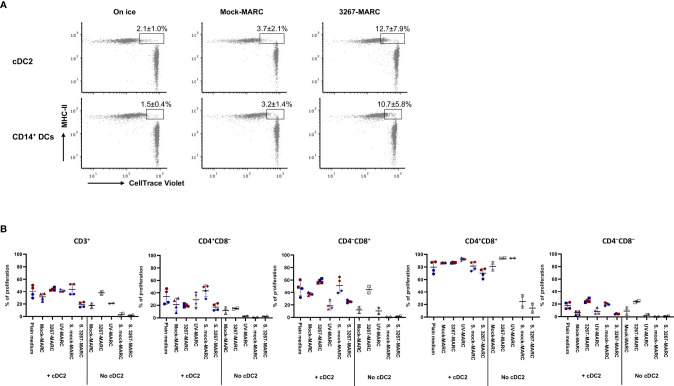
**(A)** Phagocytosis of infected MARC-145 cells by cDC2 and CD14^+^ DCs. cDC2 and CD14^+^ DCs (with MHC-II revealed by Alexa Fluor 647) were co-cultured with PRRSV1 3267 infected or mock-infected MARC-145 cells (labeled with CellTrace Violet dye) at a ratio of 1:5 at 37°C or on ice for 1 h. cDC2 and CD14^+^ DCs shown as MHC-II^+^Violet^+^ were defined as phagocytotic cells. cDC2 and CD14^+^ DCs were derived from two animals with one shown as the representative; **(B)** Comparison of T-cell proliferation by cDC2 co-cultured with infected and UV-treated MARC-145 cells. cDC2 were co-cultured for 24 h with PRRSV1 3267 infected, UV-treated, or mock-infected MARC-145 cells (3267-MARC, UV-MARC, Mock-MARC), or with plain medium or supernatants collected from infected and mock-infected MARC-145 cells (S. mock-MARC, S. 3267-MARC). Then cells were mixed with allogeneic pig PBMCs (labeled with CellTrace Violet dye) at a ratio of 1:5 and cultured for five days. Cultures in the absence of cDC2 were also included. Proliferation of CD3^+^, CD4^+^CD8α^–^, CD4–CD8α^+^, CD4^+^CD8α^+^, and CD4^–^CD8α^–^ T cells was determined by CellTrace Violet dilution (gating strategy is shown in **Supplementary Figure S3**). cDC2 were derived from two animals, as shown by the red and blue symbols. The same color means DCs were from the same pig but derived in different days.

Next, we examined some factors whereby infected cells may trigger cDC2 or CD14^+^ DCs maturation. Firstly, cDC2 and CD14^+^ DCs were exposed to the supernatants of infected MARC-145 cells (63.4 ± 3.1% of PRRSV1 N^+^ cells), but the expression of maturation molecules was not increased in either cDC2 or CD14^+^ DCs (data not shown). Then, we examined whether the activation was triggered by the phagocytosis of infected cells. As shown in **Figure 8A**, cDC2 and CD14^+^ DCs were active in phagocytosing infected MARC-145 cells or components derived from the infected cells, indicating the potential role of the phagocytic activity in DC activation. We also tested the role of apoptosis. In this case, cDC2 were co-cultivated with UV-treated MARC-145 cells (without infection) that had a proportion of apoptotic and necrotic cells (13.5 ± 2.6% of Annexin V^+^PI^–^ and 39.4 ± 1.4% of Annexin V^+^PI^+^) comparable to that of infected MARC-145 cells (63.4 ± 3.1% of PRRSV1 N^+^; 14.9 ± 1.5% of Annexin V^+^PI^–^ and 33.8 ± 2.6% of Annexin V^+^PI^+^). The results showed that exposure of cDC2 to infected or apoptotic MARC-145 cells resulted in a similar phenotypic maturation (data not shown) but differed in their ability to induce T-cell proliferation in the MLR. cDC2 co-cultured with infected MARC-145 promoted the proliferation of CD4^–^CD8^+^ and CD4^–^CD8^–^ T-cell subsets which, however, was suppressed by the use of UV-treated cells (**Figure 8B**).

## Discussion

The present study delineates the interaction of a typical PRRSV1.1 isolate (3267) with *in vitro* derived cDC1, cDC2, and CD14^+^ DCs. We show here that cDC1 and cDC2 were refractory to 3267 infection, in agreement with previous studies using lung- or tonsil-isolated cDCs (25, 33). A proportion of CD14^+^ DCs was susceptible, likely due to the expression of CD163, which is the essential receptor for PRRSV infection (34). Of note, the infection resulted in the loss of CD14 expression, as deduced from the decrease in the proportion of CD14^+^ cells in infected cultures and the fact that labeling of PRRSV N protein was mostly found in CD14^–^ cells, while, when sorted, CD14^–^ cells were refractory to the infection. In infection models by other RNA viruses such as influenza virus, HIV, Zika virus, and COVID-19, the switch of CD14 expression was also observed (35–38). In those cases, the loss of CD14 was assumed to be caused by infection-induced apoptosis but not virus-specific regulation (35). This *in vitro* derived CD14^+^ DCs were supposed to resemble moDCs. But whether this population exists *in vivo* and its roles during PRRSV infection is not known yet.

Regardless of the susceptibility, inoculation of cDC1, cDC2, and CD14^+^ DCs by PRRSV1 3267 did not induce any significant expression of the maturation-associated molecules MHC-I, MHC-II, CD80/86, or CCR7. Also, no cytokines (of the chosen panel) were produced in response to the virus, and no alterations in the endocytic and phagocytic capabilities were observed. cDC1 was a particular case because their survival time was short in the absence of poly I:C stimulation. The factors required to maintain cDC1 survival *in vitro* remain to be determined. Taken together, DCs remained immature after exposure to PRRSV1. This is compatible with the results using *ex vivo* lung DCs (25), but opposed to what is expected from the functions of DCs (12). The use of a high MOI 10 in our work makes it unlikely that DCs were not sufficiently exposed to the virus. A previous study in our group observed that the recall response against PRRSV1 3267 (IFN-γ ELISPOT) was lower than against a heterologous, less virulent strain (designated as 3262) (29) that was able to induce several cytokines in AMs, PBMCs, and GM-CSF-derived BMDCs (18, 28). It would be coherent with a lower antigen presentation ability of 3267-exposed DCs (or antigen-presenting cells in general). In the work of Bordet et al. (25), *ex vivo* DCs also remained immature after inoculation with PRRSV1 strains (moderate or high virulence). These suggest that the impairment of DC maturation can be a general feature of PRRSV1 infection; however, some other strains, such as 3262, may induce more effective responses based on the experimental data (29). Further studies are required to examine whether low-virulent strains modulate DCs distinctly. Impairment of DC maturation has been reported for other viruses such as lymphocytic choriomeningitis virus (LCMV) that evades host immune response by preventing DC maturation (39).

Although unresponsive to viral suspensions, cDC2 and CD14^+^ DCs were fully activated when co-cultivated with highly infected MARC-145 cells. Our preliminary analysis indicates phagocytosis of apoptotic or necrotic infected cells or components derived from infected cells might be the mechanism. PRRSV-infected cells were also more potent inducers of IFN-α by pDCs than cell-free virions (40). A recent study showed that cDCs and moDCs were only recruited at peak PRRSV viremia, coinciding with the induction of protective T-cell responses and the resolution of infection in pigs (24). However, other reports were unable to show such an increase (25, 33).

Based on all of these findings, it is tempting to speculate that the effective sensing of PRRSV infection by DCs is not reached until the advanced infection stage when a massive number of macrophages are infected and develop apoptosis or necrosis. This scenario would partially explain why clearance of PRRSV is delayed during infection. Current commercial PRRSV vaccines, inactivated or modified-live vaccines, are lack of satisfactory efficacy [reviewed by Hu and Zhang (41)]. The observations in our study would have implications for the development of DC-targeting vaccines, for instance, conjugating antigens to TLR ligands or targeting DC-specific endocytic receptors, *i.e.*, C-type lectin receptors DEC205, DEC209, or mannose receptor (42), to boost both the magnitude and quality of T- and B-cell responses. In the last decade, DC-targeting approaches have attracted a lot of attention to developing vaccines against HIV, cancer, and diseases that currently lack effective vaccines [as reviewed by Kastenmüller et al. (43)].

Exposure of cDC1, cDC2, or CD14^+^ DCs to the live virus did not change their antigen presentation abilities. But when cDC2 or CD14^+^ DCs were exposed to the UV-inactivated virus, the proliferation of CD4^+^CD8^–^ T cells was boosted. It is difficult to see the reasons behind it as the use of UV- inactivated virus alone did not enhance phenotypic maturation or induce secretion of the examined cytokines. Since the UV inactivation method preserves the integrity of the viral envelop glycoproteins (44), the discrepancy of results between the use of live and UV3267 is unlikely caused by virus binding or internalization but by an event later in the replication process. Alternatively, UV-damaged RNA could be processed and modified in a different way compared to untreated RNA, and as a result, it could be distinctively recognized by cytosolic receptors. In any case, the activation of TLRs or other receptors such as Retinoic acid-inducible gene (RIG)-I-like receptors (RLRs) and NOD-like receptors (NLRs) would be expected to end in cytokine production, which however was not observed with our live or UV-treated PRRSV virus. Indeed, pre-exposure to the UV-treated virus enhanced IL-10 secretion upon gardiquimod stimulation, which was consistent with a higher frequency of Tregs, at least for cDC2.

The experiments combining PRRSV1 with TLR agonists indicated that the virus may interact with TLR3 as indicated by the enhanced IFN-α secretion when cDC2 or CD14^+^ DCs were pre-exposed to the live virus. Since the virus alone (either live or inactivated) was unable to induce IFN-α, the boosting is supposed not to be a summative effect but a synergistic interaction. It is attempting to speculate that replication of the virus could start in cDC2 but was aborted before the synthesis of the viral structural proteins (as viral N protein was not detected in cDC2). Within the framework of an abortive replication, the replication activity and non-structural proteins (nsp) could be present in the cytoplasm, which may activate TLR3 signaling-associated modulators. Several proteins are known to regulate TLR3 folding, trafficking, and cleavage [see (45) for a review]. They might be candidates for interaction with the virus. But this hypothesis conflicts with the antagonizing effect of nsp4 and nsp11 in poly I:C-induced type I IFN production (46, 47).

For CD14^+^ DCs, simultaneous addition of the virus and TLR7 agonist resulted in a pan-inhibition of cytokine production. This was not associated with the susceptibility of CD14^+^ DCs because pre-exposure to the virus did not result in such effect. The pan-inhibition effect suggests that PRRSV1 may competitively suppress the response to a coinfecting pathogen that is recognized and signaled through TLR7. Swine influenza A virus (swIVA), a virus recognized by pDCs through TLR7/MyD88 signalling (48), was demonstrated to be inhibited in replication and interferon response by PRRSV that was added simultaneously (49). But why is the inhibition restricted to when the TLR7 ligand was added simultaneously with PRRSV1 but not 6 hours after PRRSV1 inoculation? Future studies aimed at delineating the precise mechanism whereby PRRSV in the coinfection or superinfection modulates a heterologous virus will be valuable for understanding the complex field cases.

For both cDC2 and CD14^+^ DCs, pre-exposure to the virus affect the proliferation of CD4^–^CD8^–^ T cells. This T-cell subset usually harbours TCR-γδ and can be divided into two subsets based on the expression of CD2 (50). Obviously, the reduction of proliferation pointed towards the CD2^+^ memory/effector subset. The role of these cells in PRRSV infection is still unknown.

In summary, our observations suggest that exposure to PRRSV1 does not induce maturation of cDC1, cDC2, or CD14^+^ DCs, but it may modify TLR-associated responses (except for cDC1). Moreover, sensing of infected cells is different from that of the free viral suspensions. But how do TLR sensor pathways coordinate the host response and the mechanisms resulting in a poor and delayed immune response to PRRSV remain to be determined.

## Data Availability Statement

The original contributions presented in the study are included in the article/**Supplementary Material**. Further inquiries can be directed to the corresponding author.

## Ethics Statement

All the cells used in this work were obtained from healthy animals euthanized according to approved procedures by the Ethics Committees of IRTA and UAB.

## Author Contributions

YL performed the experiments. YL and EM designed the experiments, analyzed the data, and wrote the manuscript. All authors contributed to the article and approved the submitted version.

## Funding

This work was funded by the Veterinary Infectious Diseases Diagnostic Laboratory of the UAB.

## Conflict of Interest

The authors declare that the research was conducted in the absence of any commercial or financial relationships that could be construed as a potential conflict of interest.
