# Supplementary material for: Interaction of Type 1 Porcine Reproductive and Respiratory Syndrome Virus With In Vitro Derived Conventional Dendritic Cells
Source: Front Immunol. 2021 Jun 2;12:674185. doi: 10.3389/fimmu.2021.674185 (PMC8221110; doi:10.3389/fimmu.2021.674185)
Supplement: Supplementary file 1 [file DataSheet_1.docx]

Supplementary Material

**Supplementary Figure S1.** Flow cytometry analysis of cell surface markers of Flt3L-derived DC cultures (unsorted) infected with PRRSV1.1 strain 3267.

1. Most of infected cells were CD14^–^. Flt3L-derived DCs (unsorted) after infection with PRRSV1.1 strain 3267 were labeled for PRRSV1 nucleocapsid (N) (X-axis) and CD14 (Y-axis) (double labeling);
2. CD14^+^ cells (X-axis) decreased in Flt3L-derived DCs (unsorted) infected by PRRSV1.1 strain 3267. A single labeling was performed. The proportion of CD14^+^ cells were marked by a red circle. Left: expression of CD14 in the cultures before infection; Middle: expression of CD14 in the mock-infected cultures after 48 h of incubation; Right: proportion of CD14^+^ cells in the infected cultures after 48 h of incubation. Channel APC (Y-axis, not occupied) was combined to well present the plot;
3. Phenotype of the infected Flt3L-derived DCs (unsorted; MOI 0.1, 48 h). Cells were stained for PRRSV1, CD14, and a third molecule (MHC-II, DEC205, CD163, CD172a, CD11R1, or CD11R3). Gating strategy: PRRSV1 N^+^ CD14 (X-axis) versus MHC-II, DEC205, CD163, CD172a, CD11R1, and CD11R3 (Y-axis).

**Supplementary Figure S2.** Uptake of dextran-FITC (upper) and phagocytic capabilities (lower, Alexa Fluor 488-labeled *S. aureus* phagocytosis) of cDC2 (left) and CD14^+^ DCs (right) after exposure to PRRSV1. For dextran-FITC (upper), results are depicted as △median fluorescence intensity (ΔMFI) by subtracting MFI of cells incubated on ice from MFI of cells incubated at 37°C (data are the means of three pigs). For the phagocytosis of *S. aureus*, results are depicted as proportion of cells positive of Alexa Fluor 488. In this case, two controls were included, cells incubated on ice and cells that were not exposed to the virus. ns = non-significant differences.

**Supplementary Figure S3.** Gating strategy of allogeneic T cell proliferation assay. PBMCs from specific-pathogen free (SPF) pigs were stained with CellTrace Violet, then mixed with allogeneic DC populations (cDC1 and cDC2, or CD14^+^ DCs) at an APC:T cell ratio of 1:5. The mixtures were cultured for 5 days at 37°C before staining with anti-CD3, anti-CD4, and anti-CD8a antibodies. Proliferation of CD4^+^CD8α^−^, CD4^−^CD8α^+^, CD4^+^CD8α^+^, and CD4^–^CD8α^–^ T cells was assessed by the percentage of cells with CellTrace Violet^low^ in the corresponding gate by flow cytometry.
